# Case Series of Chronic Inflammatory Rheumatic Disease Patients Infected by Coronavirus Disease 2019 (COVID-19)

**DOI:** 10.1155/2020/8860492

**Published:** 2020-11-12

**Authors:** Wendlassida Joëlle Stéphanie Tiendrébéogo, Fulgence Kaboré, Eric Arnaud Diendéré, Dieu-Donné Ouedraogo

**Affiliations:** ^1^Department of Rheumatology, University Hospital of Bogodogo, Ouagadougou, Burkina Faso; ^2^Department of Internal Medicine (Infectious Unity), University Hospital of Bogodogo, Ouagadougou, Burkina Faso

## Abstract

Coronavirus disease 2019 (COVID-19) is a viral infection that appeared in December 2019. The risk of infection seems to be increased in chronic inflammatory rheumatic diseases due to both immune disturbances related to the disease and treatment. In this case report, we describe the clinical features of 5 rheumatic immune disease patients with the concomitant presence of COVID-19. Among these patients, 3 had rheumatoid arthritis and 2 had systemic lupus erythematosus. Patients' age ranged between 38 and 63 years. Only one patient (SLE) had a severe subtype of COVID-19. All the patients were cured of COVID-19 and were subsequently discharged.

## 1. Introduction

Coronavirus disease 2019 (COVID-19) is a viral infection that appeared in December 2019 [1]. Its clinical manifestations depend on the degree of inflammatory response, which is linked to the host and to the virus [2]. The risk of infection seems to be increased in chronic inflammatory rheumatic diseases due to the immune disturbances associated, with the disease on one hand, and to the treatment on the other hand, mainly corticosteroids, biotherapies, and immunosuppressive treatments [3]. The health emergency imposed by this disease and the new knowledge it reveals make it necessary to specify the particularities of COVID-19 in patients with chronic inflammatory rheumatism. Few data are available on this subject.

## 2. Case Series

At date of May 31, 2020, the country (Burkina Faso) had 884 confirmed cases of Coronavirus disease 2019 since the pandemic began, which included 322 women and 562 men, and represented a sex ratio of 1.74 [4]. The diagnosis of COVID-19 was made by a positive real-time PCR testing for SARS-CoV-2 in oro and/or oropharyngeal sample. The national protocol for treatment of COVID-19 is made of hydroxychloroquine (200 mg three times daily for 10 days) and azithromycin (500 mg for Day 1 and 250 mg for Day 2 to Day 5) [5]. Early in the course of COVID-19, patients with chronic inflammatory rheumatic disease were told about the disease and barrier measures; they were also told to stop taking methotrexate or salazopyrin if ever they were a COVID-19 confirmed case. Five patients with chronic inflammatory rheumatic disease (3 rheumatoid arthritis (RA) and 2 systemic lupus erythematosus (SLE)) were among the 884 COVID-19 confirmed cases. Clinical and biological characteristics of the 5 patients are reported in Table 1.

### 2.1. Patient 1

**Figure 1:**
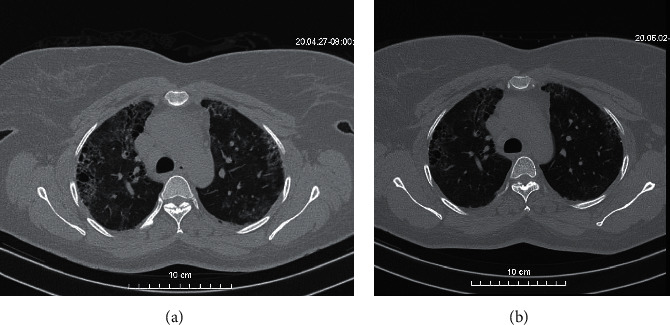
(a) Chest computed tomography (CT) scans (transverse plane) of the patient 1: frosted glass area extended central and bilateral upper lobar peripheral and fibrosis. (b) Chest computerized tomography (CT) scans (transverse plane) control of the patient 1: good evolution with almost disappearance of the frosted glass areas.

A 42-year-old woman was followed since 2017 for RA. She was treated by methotrexate (20 mg weekly), folinic acid (15 mg weekly), and methylprednisone (4 mg daily). Her disease was stable (DAS-28 CRP at 2.75). In March 2020, she developed a dry cough, severe asthenia, and fever. After 2 weeks of self-medication (antihistamines, antimalarials, antibiotics, phytomedication), she was tested positive for COVID-19 in April 2020. She then stopped taking methotrexate and replaced it by hydroxychloroquine (200 mg three times daily for 10 days) without azithromycin. The circumstances of her contamination could not be specified. She was confined at home without further treatment and was declared cured 2 weeks later after two negative controls. The evolution was made of persistence of a dry cough after the end of her confinement. A chest CT scan was then performed and showed massive bilateral interstitial pneumonitis (Figure 1(a)). To date, she is doing well without any lung disease symptoms (Figure 1(b)).

### 2.2. Patient 2

She was a 57-year-old woman with hypertension, asthma, and sleep apnea, irregularly followed in rheumatology department since January 2016 for RA. Because of digestive intolerance to methotrexate, her treatment had been changed to hydroxychloroquine (200 mg twice a day) which she admitted to be taking irregularly.

**Figure 2:**
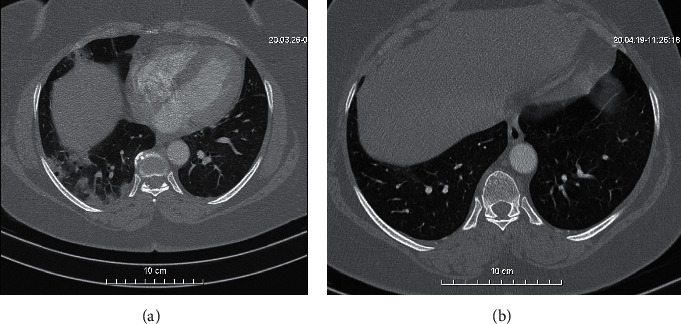
(a) Chest computed tomography (CT) scans (transverse plane) of the patient 2: multifocal nodular condensations with halo sign (peripheral frosted glass) under right posterobasal and anterobasal pleura. (b) Chest computed tomography (CT) scans (transverse plane) control of the patient 2: favorable evolution with disappearance of nodular condensations under right posterobasal pleura.

In March 2020, she attended a consultation for a dry cough that had been progressing for a few days. That cough was accompanied by asthenia, dizziness, and fever. Due to the persistence of the symptomatology despite treatment with antitussive and antibiotics, a chest CT scan was performed and showed bilateral ground glass alveolo-interstitial pneumonitis referring to COVID-19 (Figure 2(a)). A nasopharyngeal sample confirmed Coronavirus 2019 infection. She was hospitalized and treated by hydroxychloroquine and azithromicin, intravenous corticosteroids, and salbutamol. Her condition did not require admission to intensive care unit. She was declared cured after two COVID-19 negative controls and was discharged from hospital in April 2020. A follow-up chest CT scan was performed and showed an almost complete regression of the parenchymal signs of COVID-19 (Figure 2(b)).

### 2.3. Patient 3

He was a 63-year-old man followed for RA for five years on methotrexate (15 mg per week) and folinic acid (10 mg weekly) and who also had heart disease and keratitis.

Four days earlier, due to deep asthenia, flu syndrome secondary to contact with a colleague who has been tested positive for COVID-19, a nasal swab was taken. The result available on March 20, 2020, confirmed a COVID-19 infection. Treatment with methotrexate was suspended. He was hospitalized and treated by hydroxychloroquine and azithromicin. His condition did not require admission into intensive care unit. Two weeks later, two successive tests were negative, confirming his recovery.

### 2.4. Patient 4

She was a 38-year-old woman with SLE (involving skin and joint) for three years, treated by hydroxychloroquine (200 mg twice daily). In March, she attended a consultation for headaches and arthromyalgia. Because of the persistence of the symptomatology, a nasopharyngeal swab was taken and confirmed the diagnosis of COVID-19. The evolution was favorable with treatment by hydroxychloroquine and azithromycin according to the national protocol.

### 2.5. Patient 5

She was a 42-year-old woman, followed for about 10 years for SLE with chronic renal failure and nephrotic syndrome and treated by monthly boluses of cyclophosphamide relayed by Azathioprine. In March 2020, she attended a medical consultation for respiratory distress syndrome. Nasopharyngeal swabbing confirmed the diagnosis of COVID-19. Due to the severity of the symptomatology, she was admitted to intensive care unit and treated by intravenous corticosteroids, orotracheal intubation, and oxygen therapy and was discharged from hospital.

## 3. Discussion

Since the advent of COVID-19, one of the concerns of rheumatologists has been the risk of intensive care unit admission and excess mortality in patients with chronic inflammatory rheumatic disease [6–8]. Few series have been published [9–12]. The incidence of COVID-19 during chronic inflammatory rheumatic disease has not been studied in our series; however, it does not seem to be different from that of the general population [10, 12]. We report a series of five patients (3 RA and 2 SLE) infected by COVID-19.

In patients with RA, the clinical feature of COVID-19 was moderate with recovery of the disease without admission to intensive care. Four of the five patients of Cheng et al. series, although a higher mean age, also had a favorable outcome without admission to the intensive care unit [9]. While waiting for larger series or a meta-analysis, we have the impression that rheumatoid arthritis is not a risk factor for admission to intensive care or for mortality during COVID-19 regardless of the background treatment (csDMARD, bDMARD, and tsDMARD). Patients 1 and 2 had a CT scan showing bilateral ground glass interstitial pneumonitis that regressed after healing as reported by Song et al. in their case report [11].

Few series have been reported on SLE cases with COVID-19. Of the two patients with SLE in our series, patient 5 had a severe subtype of COVID-19 that required orotracheal intubation. In a series by Wallace et al. about 5 SLE, four patients (80%) were hospitalized for COVID-19; three (60%) required invasive ventilation; and one (20%) died of the disease [13]. According to Sawalha et al., patients with SLE are at risk of developing severe COVID-19 due to overexpression of the angiotensin-converting enzyme receptor [14]. It should be noted, however, that patient 5 had significant comorbidities such as heart disease, chronic renal failure, and nephrotic syndrome known to be risk factors for admission to intensive care unit [15].

## 4. Conclusion

Infection with Sars-Cov 2019 does not appear to be a poor prognosis in patients with rheumatoid arthritis. Patients with SLE would be at risk of severe COVID-19, especially if the patient has mutivisceral SLE damage. However, definitive conclusions must await the results of registries or meta-analyses before any definitive conclusions can be drawn.

## Figures and Tables

**Table 1:** Clinical characteristics and laboratory test of 5 patients.

	Patient 1	Patient 2	Patient 3	Patient 4	Patient 5
Rheumatic diseases	RA	RA	RA	SLE	SLE
*Clinical characteristics*					
Cough	Yes	Yes	Yes	No	Yes
Fever	Yes	Yes	Yes	No	Yes
Rhinorrhea	No	No	Yes	No	No
Anosmia	No	No	Yes	No	No
Ageusia	No	No	Yes	No	No
Nasal obstruction	No	No	Yes	No	No
Arthralgia and myalgia	Yes	No	No	Yes	No
Headache	Yes	Yes	Yes	Yes	Yes
Asthenia	Yes	Yes	Yes	No	No
Vomiting	No	No	No	No	No
Diarrhea	No	No	No	No	No
Temperature	38.7	36.9	38.9	37.1	38.4
Blood pressure	130/80	150/100	120/75	152/110	98/69
Oxygen saturation	98	97	98	95	78
Respiratory frequency	Normal	Normal	Normal	Normal	28
Heart rate	Normal	100	Normal	Normal	130
Clinical subtype	Moderate	Moderate	Moderate	Moderate	Severe

*Laboratory parameters*					
Hemoglobin (g/dL)	12.5	12.3	13.7	13.2	12.4
White blood cells (mm^3^)	6800	6100	9030	5560	19910
Segment neutrophiles (mm^3^)	4864	4026	7040	2520	18180
Lymphocytes (mm^3^)	1515	1769	1250	2580	1040
Plaquets (mm^3^)	271000	254000	324000	172000	333000
C-reactive protein (mg/l)	190.29	26	171.34	10.05	376.28
AST (IU/L)	30	22	36	10.63	49.91
ALT (IU/L)	31	25	41	13.06	47.02
Urea (mmol/L)	3.47	3.75	3.68	1.71	6.85
Creatinine (micromoles/L)	69.92	84	76.8	64.5	137.5
Sodium (mmol/L)	146	146	133	144	125
Potassium (mmol/L)	4.25	4.33	3.3	4.1	2.7
Calcemia (mmol/L)	2.22	2.28	2.42	2.58	2.4

RA, rheumatoid arthritis; SLE, systemic erythematosus lupus; AST, aspartate transaminase; ALT, alanine transaminase.
